# Analysis of the prognostic significance of solute carrier (SLC) family 39 genes in breast cancer

**DOI:** 10.1042/BSR20200764

**Published:** 2020-08-13

**Authors:** Limei Liu, Jiaomin Yang, Chao Wang

**Affiliations:** 1 Department of Keratonosus, Weifang Eye Hospital, Weifang 261053, Shandong, China; 2 Department of Laboratory Medicine, Weifang Yidu Central Hospital, Qingzhou 262500, Shandong, China; 3 Department of Pathogen Biology, Weifang Medical University, Weifang 261053, Shandong, China

**Keywords:** breast cancer, prognosis, SLC39A7, SLC39A, ZIP7

## Abstract

**Background:** Breast cancer (BC) is the most common malignancy in females and remains a main cause of cancer-associated death worldwide. The solute carrier (SLC) groups of membrane transport proteins, which control the influx of zinc, participate in ranging of physiological processes and may provide novel therapeutic targets of cancers. However, the prognostic values of individual SLC family 39 (SLC39A) genes in patients with BC are not clarified.

**Materials and Methods:** The mRNA expression of SLC family 39 genes in BC was evaluated by using the UALCAN database. The prognostic values of overall survival (OS) of SLC family 39 genes in patients with BC were investigated by Kaplan–Meier plotter. The survival analysis of cells was determined by Project Achilles.

**Results:** The analytic results suggested that SLC39A1, SLC39A3, SLC39A4, SLC39A5, SLC39A6, SLC39A7, SLC39A9, SLC39A10, SLC39A11 and SLC39A13 were significantly up-regulated in BC tissues compared with normal breast tissues. However, SLC39A8 and SLC39A14 were expressed higher in normal tissues than in BC tissues. High expression of SLC39A2, SLC39A3, SLC39A4, SLC39A5, SLC39A7, SLC39A12 and SLC39A13 was significantly associated with worse OS in patients with BC. In contrast, high mRNA levels of SLC39A6 and SLC39A14 indicated favorable OS. Through subgroup analysis, all abnormal expressed SLC family members were correlated with prognoses of patients with specific BC. Moreover, SLC39A7 was associated with proliferation and cloning of BC.

**Conclusions:** Our results suggested that SLC family 39 members were promising prognostic biomarkers of BC. The SLC39A7 played a key role in growth and survival of BC cells.

## Introduction

Breast cancer (BC) is the most common malignancy in females and remains a main cause of cancer-associated death worldwide [1]. In recent years, whole-genome gene expression profiling based on expression of estrogen receptor alpha (ERα), progesterone receptor (PR) or the human epidermal growth factor receptor 2 (HER2) has allowed to evaluate the prognosis and apply targeted treatment strategy of patients with BC, but long-term survival is still not optimistic due to frequent recurrence and metastasis [2–4]. Therefore, identifying new diagnostic biomarkers and or therapeutic targets for the BC management is urgent to be accomplished.

The solute carrier (SLC) groups of membrane transport proteins, which have 65 families, participate in ranging of physiological processes and may provide novel therapeutic targets for human malignances [5,6]. The concentration of zinc in cells is tightly controlled by two groups of zinc transport proteins: ZnT transporters (SLC30A or zinc exporter) and ZIP channels (SLC39A, Zrt- and Irt-like proteins, or zinc importers) [7]. By transferring zinc from the extracellular space or intracellular stores, ZIP channels increase the cytosolic zinc level, whereas ZnT transporters facilitate zinc transport in the opposite direction [8]. ZIP channels have four subfamilies including: subfamily I (ZIP9), subfamily II (ZIP1–3), LIV-1 subfamily (ZIP4–8, 10, and 12–14), and gufA (ZIP11) [9].

Lines of studies have reported the key functions of SLCA39 genes in different kinds of malignances, like esophageal carcinoma, lung cancer, colorectal cancer, hepatocellular carcinoma and prostate cancer [10–15]. In addition, studies have demonstrated that parts of SLCA39 genes may be related to patient’s survival in some types of cancer and could serve as cancer diagnostic or prognostic biomarkers. For example, by comparing endoscopic ultrasound guided fine needle aspiration (EUS-FNA) and surgical specimens, Xu et al*.* have found that SLC39A4 is a potential prognostic and diagnostic marker in pancreatic cancer [15]. Ding et al. have evaluated the prognostic values of all SLCA39 genes and gastric cancer [16]. To our knowledge, study regarding the expression pattern and prognostic values of the total SLCA39 genes and BC is still absent.

In the present study, we first evaluated the significance of SLCA39 genes expression and prognosis in BC by using comprehensive bioinformatics analysis of the clinical indicators and survival data in several large online databases. Then, the growth affected evaluation was performed to identify the key gene of SLCA39 genes in BC.

## Methods and materials

### Ualcan database

UALCAN is a web analysis tool that provides a comprehensive cancer transcriptome data come from TCGA database (http://ualcan.path.uab.edu/) [17]. All the mRNA expression of SLC39A genes expression in BC tissue and in corresponding normal breast tissue was evaluated by using UALCAN database. In addition, UALCAN provides the analysis of protein expression of CPTAC. The comparison of SLC39A7 expression between BC tissue and normal breast tissues in protein level was performed in UALCAN database.

### Kaplan–Meier plotter

Kaplan-Meier plotter is capable to assess the effect of 54000 genes on survival in 21 cancer types (http://kmplot.com/analysis/index.php?p=background) [18]. In order to determine the potential prognostic values of SLC39A genes in BC, the relationship between mRNA expression levels of SLC39A genes and overall survival (OS) of patients with BC was analyzed by using the Kaplan–Meier plotter. Next, the OS of SLC39A genes associated with relatively clinicopathological features were analyzed by this database. Furthermore, the prognostic values between SLC39A7 on protein level and patients with BC were also analyzed by Kaplan–Meier plotter. All analysis used automatically selecting best cutoff. The follow up threshold is set as maximum.

### cBioportal for cancer genomics

cBioportal for Cancer Genomics (http://www.cbioportal.org/) is a comprehensive web to analysis the data onto TCGA. We obtained the mRNA expression data and clinical data of patients with BC (TCGA-BRCA) from TCGA in this Web.

### Gene set enrichment analysis of SLC39A7

GSEA was performed to annotate the Hallmark effector gene sets associated with SLC39A7 mRNA expression of the TCGA-BRCA dataset [19]. GSEA software was obtained from the Broad Institute (http://www.broad.mit.edu/gsea).

### Scores of project achilles

Project Achilles systematically identifies and catalogs gene essentiality across hundreds of genomically characterized cancer cell lines [20]. Data evaluating the importance of SLCA39 genes for cell survival of BC were downloaded from Depmap portal (https://depmap.org/portal).

### Cell lines and reagents

MCF7 cell line was a gift from Prof. Xiaoping Sun (Wuhan University, Wuhan, China). MDA-MB-468 was kindly provided from Dr. Ye Wang (Qingdao Hospital of Traditional Chinese Medicine, Qingdao, China). All cell lines were grown in DMEM media (HyClone; U.S.A.) containing 10% FBS (Gibco; U.S.A.) and 1% antibiotic (penicillin/ streptomycin, Sigma; U.S.A.), and maintaining at humidified atmosphere of 5% CO_2_ and 37°C.

### Cell transfection

MCF7 and MDA-MB-468 cells were transfected with small interfering RNA (siRNA) targeting SLC39A7 (5′-ACAAGAAAGGCAACAAUUCCAGAAUUGUUGCCUUUCUUGUCG-3′, GenePharma, Co., Ltd.) or a non-specific control using X-treme GENE HP DNA Transfection Reagent (Roche Diagnostics) according to the manufacturer’s instructions.

### Cell viability assay

MCF7 and MDA-MB-468 cells (1 × 10^4^ cells/well) were seeded in 96-well plates, transfected and incubated at 37°C. At indicated time after transfection (0.5, 1.5, 2.5, 3.5 d), 10 μl Cell Counting Kit 8 reagents (Dojindo Molecular Technologies; Japan) were added in the cells. Then cells were incubated at 37°C for 1 h in the dark. The luminescence value was detected by using an ELx800 microimmunoanalyser (BioTek Instruments; U.S.A.) [21].

### Colony-formation assay

After transfection, 500 MCF7 and MDA-MB-468 cells were placed in 35 mm culture dishes and incubated with DMEM media (HyClone; U.S.A.) containing 10% FBS (Gibco; U.S.A.) at 37°C for 14 days. Then cells were washed and treated with 0.5% crystal violet-glutaraldehyde solution (0.6%, v/v) for 1min. Cell colony was photographed after washing with PBS [22].

### Western blot analysis

The detailed method of Western blot assay was described in our previous publication [23]. Briefly, MCF7 and MDA-MB-468 cells were transfected with siRNA-SLC39A7 or the control siRNA for 48 h. Cells were rinsed, harvested and dissolved in RIPA lysis buffer (Beyotime Institute of Biotechnology, China) with 0.5% cocktail protease inhibitor (Roche Diagnostics). Cell lysates were incubated on ice for 10 min, collected and sonicated for 15 s. Protein concentration was measured by BCA assay (BioRad Laboratories, U.S.A.). Protein was balanced, mixed with 5× loading buffer [Chunfeng Lv, China] and boiled for 5 min. Lysates were run 10% SDS-PAGE gels then transferred to PVDF membrane. After blocking with 5% milk in PBS (0.01% Tween 20) for 1 h, membranes were hybridized to primary antibodies overnight at 4°C. The primary antibodies used were as follows: GAPDH (cat no. 5174; 1:2000; Cell Signaling Technology, Inc; U.S.A.); SLC39A7 (cat no. ab254566; 1:1000; Abcam, U.K.).

### Statistical analysis

Experiments were repeated triply, and results were shown as mean ± standard deviation (SD). One way ANOVA followed with Newman–Keuls post analysis was used to analysis the comparison between more than two groups. Student’s *t* test was applied for determining significance of two comparative groups. *P*-value <0.05 was considered as statistical significance. All statistical analyses were analyzed by using SPSS for Windows version 12.0.

## Results

### Relative mRNA expression SLC39A genes in BC

**Figure 1:**
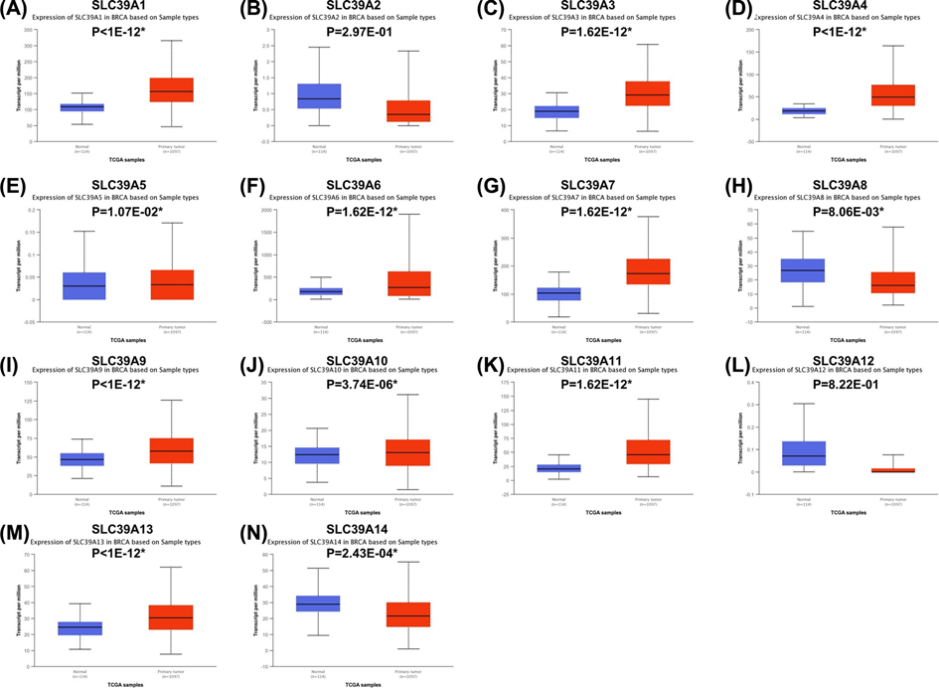
Expression of mRNA of SLC39A genes varied in primary tumor and in corresponding normal tissues in patients with breast cancer by using Uaclan database (**A–N**) SLC39A1 – SLC39A14, *P<0.05.

To explore the roles of SLC39A genes expression of BC, expression data from TCGA database onto 14 SLC39A genes in breast invasive carcinoma patients were analyzed by using UALCAN. As shown in Figure 1, expression of SLC39A1, SLC39A3, SLC39A4, SLC39A5, SLC39A6, SLC39A7, SLC39A9, SLC39A10, SLC39A11 and SLC39A13 was significantly higher in cancer tissue than which in normal tissue; however, SLC39A8 and SLC39A14 were expressed higher in normal tissue. The expression of SLC39A2 and SLC39A12 between cancer and normal tissue showed no significant difference.

### SLC39A genes have high prognostic values in BC patients using Kaplan–Meier plotter

**Figure 2:**
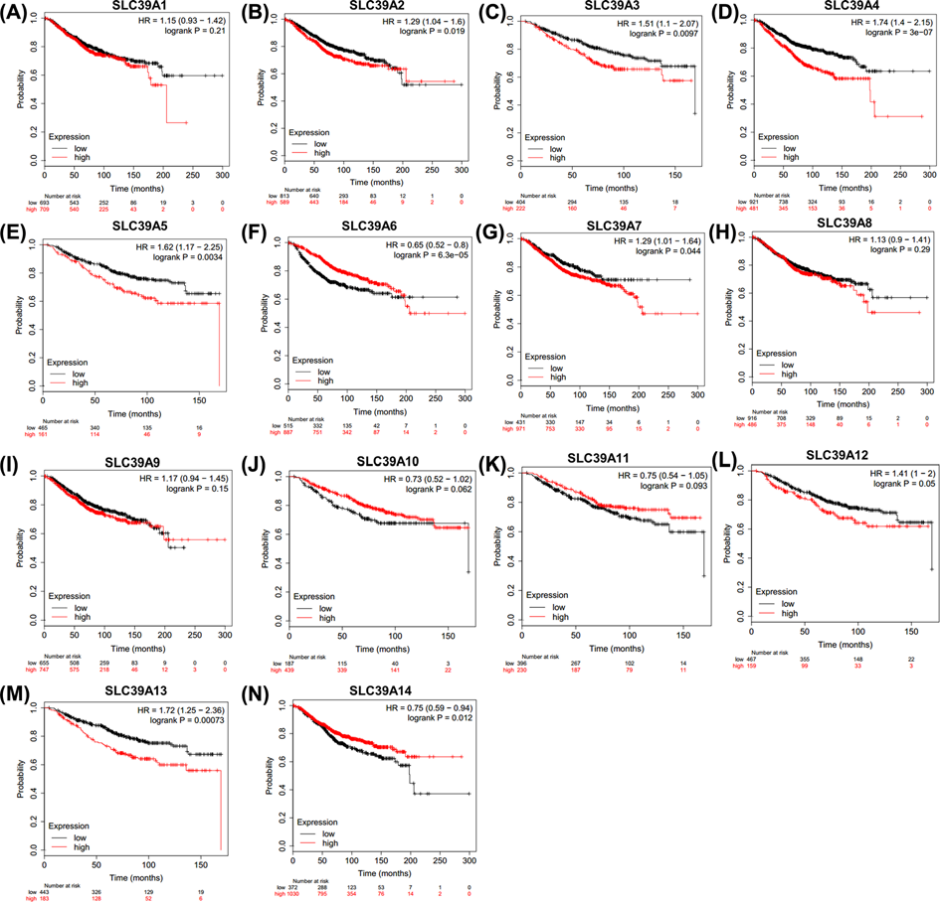
Overall survival of SLC39A genes in patients with breast cancer by using Kaplan–Meier plotter (**A–N**) SLC39A1 – SLC39A14.

Then, the potential prognostic values of SLC39A genes in BC were investigated by using Kaplan–Meier plotter. As shown in Figure 2, most of those genes showed positive relationships between high expression and significant worse OS in patients with BC, including SLC39A2 [HR = 1.29(1.04–1.6), *P*=0.019], SLC39A3 [HR = 1.51(1.1–2.07), *P*=0.0097], SLC39A4 [HR = 1.74(1.4–2.15), *P*=3E-07], SLC39A5 [HR = 1.62(1.17–2.25), *P*=0.0034], SLC39A7 [HR = 1.29(1.01–1.64), *P*=0.044], SLC39A12 [HR = 1.41(1-2), *P*=0.05] and SLC39A13 [HR = 1.72(1.25–2.36), *P*=0.00073]. In contrast, high mRNA levels of SLC39A6 [HR = 0.65(0.52–0.8), *P*=6.3E-05] and SLC39A14 [HR = 0.75(0.59–0.94), *P*=0.012] showed favorable OS. Other genes including SLC39A1 [HR = 1.15(0.93–1.42), *P*=0.21], SLC39A8 [HR = 1.13(0.9–1.41), *P*=3E-07], SLC39A9 [HR = 1.17(0.94–1.45), *P*=0.15], SLC39A10 [0.73(0.52–1.02), *P*=0.062] and SLC39A11 [HR = 0.75(0.54–1.05), *P*=0.093] were not associated with the OS of BC patients.

To further to understand the significant roles of SLC39A genes in prognoses of patients with BC, the OS between SLC39A genes expression and various clinicopathological features based on intrinsic sub-types, lymph node status, differentiation and endocrine therapy status of patients with BC was investigated. According to the expression of ER, PR and HER2, patients with BC were divided into four molecular subtypes: Basal (triple negative), Luminal A (LumA, ER/PR +, HER2 -), Luminal B (LumB, ER/PR +, HER2 +/-) and HER2 (ER/PR -, HER2 +).

As shown in Table 1, SLC39A2, SLC39A3 and SLC39A10 high expression were significantly related with worse OS in patients with Basal BC. But no significant association was found between other SLC39A genes and OS in patients with Basal BC. High mRNA expression of SLC39A4, SLC39A7 and SLC39A13 was also found to be correlated with worse OS in patients with LumA BC. In contrast, SLC39A12 and SLC39A14 high expression indicated better OS in patients with LumA BC. For patients with LumB BC, high mRNA expression of SLC39A1, SLC39A3, SLC39A4, SLC39A9, SLC39A11 and SLC39A13 was found to be associated with worse OS. SLC39A8 mRNA high expression suggested a better OS. For patients with HER2 BC, SLC39A1, SLC39A5, SLC39A9 and SLC39A13 high expression suggested a worse OS, but SLC39A6 and SLC39A14 high expression suggested a benefit OS.

**Table 1:** Overall survival between SLC family 39 genes expression and intrinsic sub-types of breast cancers patients

Genes	Basal			LumA			LumB			HER2		
	Cases	HR	*P*	Cases	HR	*P*	Cases	HR	*P*	Cases	HR	*P*
SLC39A1	241	0.71 (0.44–1.16)	0.17	611	0.71 (0.49–1.03)	0.066	431	1.53 (1.03–2.29)	0.034*	117	2.41 (1.1–5.27)	0.023*
SLC39A2	241	2.5 (1.52–4.11)	0.0002*	611	1.18 (0.83–1.69)	0.36	431	1.33 (0.92–1.93)	0.13	117	0.78 (0.4–1.51)	0.46
SLC39A3	153	3.33 (1.75–6.32)	9.4e–5*	271	0.71 (0.43–1.18)	0.18	129	2.31 (1.17–4.56)	0.013*	73	1.95 (0.87–4.36)	0.097
SLC39A4	241	1.61 (0.89-2.91)	0.11	611	2.01 (1.41–2.81)	8.1e-5*	431	1.53 (1.06–2.22)	0.022*	117	0.66 (0.33–1.32)	0.24
SLC39A5	153	1.84 (0.97–3.49)	0.058	271	0.64 (0.38–1.07)	0.086	129	1.6 (0.8–3.22)	0.18	73	3.96 (1.78–8.83)	0.0003*
SLC39A6	241	1.59 (0.59–2.65)	0.073	611	0.84 (0.59–1.2)	0.34	431	1.31 (0.91–1.89)	0.15	117	0.53 (0.25–1.12)	0.092
SLC39A7	241	0.68 (0.41–1.12)	0.13	611	1.48 (1.03–2.11)	0.033*	431	1.36 (0.91–2.35)	0.13	117	0.59 (0.28–1.22)	0.15
SLC39A8	241	0.8 (0.47–1.36)	0.42	611	1.32 (0.93–1.89)	0.12	431	0.66 (0.45–0.97)	0.033*	117	1.92 (0.91–4.07)	0.083
SLC39A9	241	1.39 (0.85–2.27)	0.19	611	0.71 (0.5–1.02)	0.063	431	1.65 (1.08–2.53)	0.02*	117	2.6 (1.09–6.25)	0.026*
SLC39A10	153	2.21 (1–4.48)	0.044*	271	0.76 (0.46–1.27)	0.29	129	1.66 (0.85–3.26)	0.14	73	0.34 (0.15–0.75)	0.0053*
SLC39A11	153	1.64 (0.86–3.13)	0.13	271	0.62 (0.37–1.05)	0.072	129	2.21 (1.42–4.39)	0.019*	73	0.5 (0.21–1.17)	0.1
SLC39A12	153	1.47 (0.77–2.79)	0.24	271	0.56 (0.33–1.93)	0.022*	129	0.6 (0.3–1.22)	0.15	73	0.67 (0.3–1.46)	0.31
SLC39A13	153	1.79 (0.93–3.47)	0.078	271	1.78 (1.08–2.94)	0.023*	129	2.04 (0.99–4.19)	0.047*	73	5.2 (1.55–17.37)	0.0028*
SLC39A14	241	0.75 (0.45–1.26)	0.28	611	0.67 (0.47–0.97)	0.033*	431	0.82 (0.56–1.18)	0.28	117	0.45 (0.21–0.98)	0.04*

* The *P* value means statistical significant.

The results of association between SLC39A genes expression and OS in patients with BC based on lymph node status were shown in Table 2. For patients with positive lymph node metastasis, SLC39A2, SLC39A3, SLC39A7, SLC39A8, SLC39A12 and SLC39A13 high expression suggested a worse OS, but SLC39A10 high expression suggested a benefit OS. For patients have no lymph node metastasis, high mRNA expression of SLC39A4 and SLC39A12 was also found to be correlated with worse OS. In contrast, SLC39A6 and SLC39A14 high expression indicated better OS.

**Table 2:** Overall survival between SLC family 39 genes expression and lymph node status of breast cancers patients

Genes	Lymph node positive			Lymph node negative		
	Cases	HR	*P*	Cases	HR	*P*
SLC39A1	313	1.24 (0.83–1.87)	0.29	594	1.34 (0.92–1.95)	0.12
SLC39A2	313	1.64 (1.02–2.66)	0.04*	594	1.22 (0.84–1.77)	0.28
SLC39A3	177	2.28 (1.22–4.24)	0.0075*	122	1.61 (0.61–4.28)	0.33
SLC39A4	313	1.56 (1.06–2.3)	0.023*	594	1.62 (1.12–2.36)	0.01*
SLC39A5	177	1.86 (1.08–3.2)	0.023*	122	0.43 (0.17–1.06)	0.06
SLC39A6	313	0.74 (0.49–1.2)	0.16	594	0.54 (0.37–0.78)	0.0009*
SLC39A7	313	1.68 (1.11–2.54)	0.013*	594	0.79 (0.55–1.15)	0.23
SLC39A8	313	1.56 (1.06–2.3)	0.023*	594	1.25 (0.85–1.82)	0.25
SLC39A9	313	1.46 (0.99–2.16)	0.057	594	0.75 (0.51–1.11)	0.15
SLC39A10	177	0.4 (0.24–0.69)	0.0007*	122	2.37 (0.96–5.85)	0.053
SLC39A11	177	0.68 (0.39–1.19)	0.18	122	0.48 (0.19–1.22)	0.11
SLC39A12	177	1.78 (1.01–3.14)	0.044*	122	2.69 (1.09–6.68)	0.026*
SLC39A13	177	2.8 (1.65–4.73)	6.1e–5*	122	1.87 (0.54–6.42)	0.31
SLC39A14	313	0.68 (0.44–1.05)	0.079	594	0.68 (0.47–0.98)	0.039*

* The *P* value means statistical significant.

According to the cell differentiation status, BC was divided into three subtypes, including well, moderate and poor differentiation. The results are shown in Table 3. For patients with well differentiation BC, high mRNA expression of SLC39A2 and SLC39A14 were found to be associated with worse OS. However, SLC39A1 and SLC39A5 mRNA high expression suggested a better OS. SLC39A4 high expression was significantly related with worse OS in patients with moderately differentiated BC, but SLC39A6 and SLC39A14 high expression showed benefit OS. For patients with poor differentiated BC, SLC39A1, SLC39A2, SLC39A3, SLC39A4 and SLC39A13 mRNA high expression indicated a poor prognosis of OS, whereas high expression of SLC39A11 suggested a better OS.

**Table 3:** Overall survival between SLC family 39 genes expression and differentiation of breast cancers patients

Genes	Well differentiated			Moderately differentiated			Poorly differentiated		
	Cases	HR	*P*	Cases	HR	*P*	Cases	HR	*P*
SLC39A1	161	0.23 (0.05–1)	0.032*	387	1.33 (0.86–2.07)	0.2	503	1.72 (1.23–2.41)	0.0015*
SLC39A2	161	2.39 (0.92–6.2)	0.064	387	1.36 (0.81–2.26)	0.24	503	1.42 (1.02–1.97)	0.0035*
SLC39A3	26	1.34 (0.87–2.09)	0.21	64	1.64 (0.44–6.08)	0.46	204	1.71 (1.01–2.88)	0.043*
SLC39A4	161	1.61 (0.65–3.97)	0.29	387	2.04 (1.33–3.13)	0.0008*	503	1.46 (1.03–2.06)	0.032*
SLC39A5	26	0.12 (0.01–1.39)	0.045*	64	2.85 (0.92–8.83)	0.058	204	1.69 (0.98–2.91)	0.058
SLC39A6	161	0.68 (0.28–1.64)	0.38	387	0.59 (0.39–0.91)	0.016*	503	0.8 (0.58–1.11)	0.18
SLC39A7	161	1.99 (0.58–6.88)	0.27	387	1.34 (0.86–2.08)	0.19	503	1.42 (0.97–2.09)	0.071
SLC39A8	161	3.5 (0.8–15.31)	0.077	387	0.71 (0.45–1.13)	0.15	503	1.27 (0.91–1.76)	0.16
SLC39A9	161	0.49 (0.2–1.22)	0.12	387	1.25 (0.8–1.96)	0.33	503	1.35 (0.91–2)	0.13
SLC39A10	26	0.38 (0.18–1.31)	0.16	64	0.4 (0.13–1.25)	0.1	204	0.59 (0.35–1.01)	0.054
SLC39A11	26	2.52 (0.23–27.9)	0.43	64	0.41 (0.11–1.51)	0.16	204	0.55 (0.31–0.96)	0.034*
SLC39A12	26	5.59 (0.51–61.82)	0.11	64	2.02 (0.64–6.42)	0.22	204	1.5 (0.89–2.51)	0.12
SLC39A13	26	0.32 (0.03–3.6)	0.33	64	2.43 (0.78–7.58)	0.11	204	2.62 (1.57–4.36)	0.0001*
SLC39A14	161	2.67 (0.98–7.22)	0.045*	387	0.52 (0.34–0.81)	0.003*	503	0.8 (0.57–1.13)	0.2

* The *P* value means statistical significant.

Endocrine therapy is an important method of BC treatment according to the intrinsic sub-types. As shown in Table 4, for patients treated without endocrine therapy, mRNA high expression of SLC39A3, SLC39A7, SLC39A11 and SLC39A13 indicated poor prognoses of OS, but high expression of SLC39A6 and SLC39A9 suggested better OS. For those patients combined endocrine therapy and other treatment, SLC39A7, SLC39A9 and SLC39A13 high expression were associated with worse OS, but SLC39A5 and SLC39A10 high expression were associated with better prognoses of OS. High expression of SLC39A4, SLC39A7 and SLC39A9 were correlated with poorer OS, and SLC39A5 high expression suggested a benefit OS.

**Table 4:** Relapse free survival between SLC family 39 genes expression and endocrine therapy status of breast cancers patients

Genes	Excluded			Included			Tamoxifen only		
	Cases	HR	*P*	Cases	HR	*P*	Cases	HR	*P*
SLC39A1	1606	0.88 (0.73–1.07)	0.2	1064	0.77 (0.61–0.99)	0.039*	809	0.75 (0.56–1.01)	0.054
SLC39A2	1606	0.89 (0.75–1.06)	0.19	1064	0.83 (0.65–1.07)	0.14	809	1.31 (0.98–1.75)	0.066
SLC39A3	275	1.62 (1.04–2.53)	0.03*	335	0.62 (0.38–1.02)	0.059	161	2.06 (0.81–5.23)	0.12
SLC39A4	1606	1.5 (1.25–1.8)	1.4e–5*	1064	1.54 (1.2–1.96)	0.0005*	809	1.49 (1.11–1.98)	0.0067*
SLC39A5	275	1.44 (0.91–2.29)	0.12	335	0.47 (0.3–0.76)	0.0014*	161	0.31 (0.12–0.82)	0.012*
SLC39A6	1606	0.66 (0.55–0.79)	9.7e–6*	1064	0.85 (0.65–1.12)	0.24	809	0.76 (0.54–1.07)	0.11
SLC39A7	1606	1.4 (1.17–1.68)	0.0003*	1064	1.52 (1.19–1.94)	0.0006*	809	1.68 (1.21–2.33)	0.0016*
SLC39A8	1606	0.86 (0.72–1.02)	0.084	1064	0.77 (0.58–1.01)	0.057	809	0.75 (0.54–1.04)	0.089
SLC39A9	1606	0.82 (0.69–0.98)	0.027*	1064	1.56 (1.13–2.16)	0.0068*	809	1.74 (1.18–2.58)	0.0048*
SLC39A10	275	1.3 (0.81–2.08)	0.28	335	0.48 (0.27–0.84)	0.0083*	161	1.35 (0.54–3.34)	0.52
SLC39A11	275	2.38 (1.54–3.69)	5.9e–5*	335	1.91 (1.12–3.26)	0.016*	161	2.44 (0.81–7.34)	0.1
SLC39A12	275	1.36 (0.88–2.11)	0.17	335	1.46 (0.92–2.33)	0.11	161	1.86 (0.67–5.2)	0.23
SLC39A13	275	2.01 (1.29–3.14)	0.0018*	335	1.59 (1–2.54)	0.049*	161	0.59 (0.24–1.47)	0.25
SLC39A14	1606	0.89 (0.74–1.07)	0.21	1064	0.87 (0.65–1.16)	0.33	809	1.2 (0.89–1.61)	0.22

* The *P* value means statistical significant.

### SLC39A7 is a key gene for survival of BC cells

**Figure 3:**
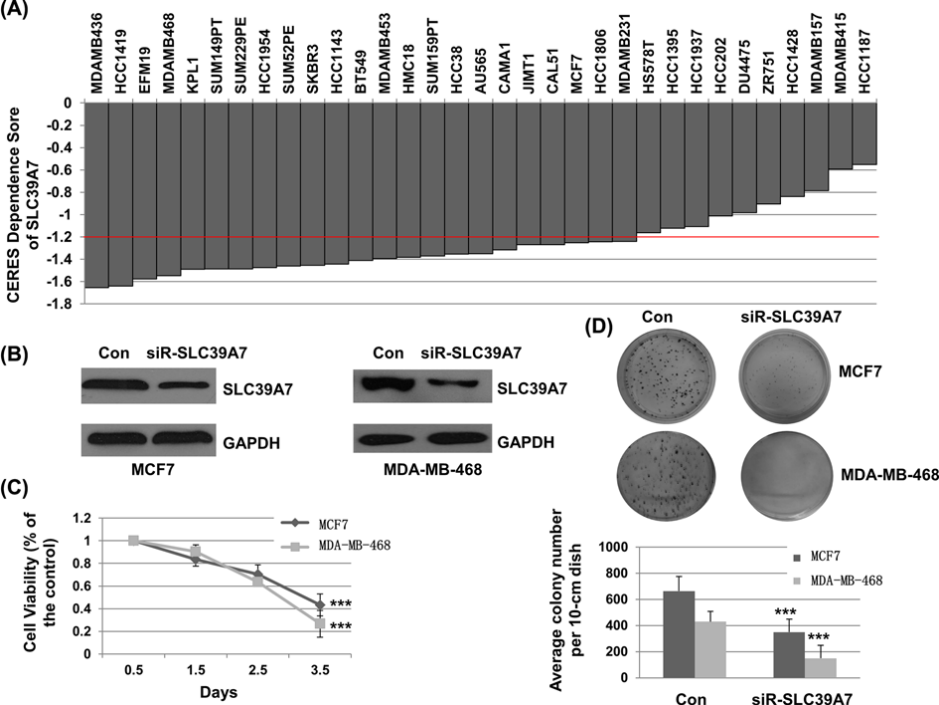
SLC39A7 played a vital role in cell survival of breast cancer (**A**) CERES dependence score of SLC39A7 in difference breast cancer cell lines, CERES score approach to 0 means the gene is not an essential gene for cell survival, while score approach to -1 means the gene is an essential gene. (**B**) MCF7 and MDA-MB-468 cells were transfected with siRNA-SLC39A7 or a control siRNA for 48 h. Cells were harvested and subjected for Western blotting. (**C**) MCF7 and MDA-MB-468 cells were seeded, transfected and incubated. At indicated time after transfection (0.5, 1.5, 2.5, 3.5 d), cell viability was detected by a CCK-8 assay. (**D**) After transfection, MCF7 and MDA-MB-468 cells were placed and incubated with standard medium at 37°C for 14 days. Then, cells were washed and treated with crystal violet-glutaraldehyde solution. The photographs and the detailed column chart were given; ***P<0.001.

To determine whether SLC39A genes are essential for cell survival of BC, we investigated the data from Project Achilles. CERES dependence score was obtained from Depmap. As shown in Figure 3A, when knocking down on SLC39A7 gene with CRISPR–Cas9 system, CERES scores of 27 BC cell lines were less than -1 (MDAMB436, HCC1419, EFM19, MDAMB468, KPL1, SUM149PT, SUM229PE, HCC1954, SUM52PE, SKBR3, HCC1143, BT549, MDAMB453, HMC18, SUM159PT, HCC38, AU565, CAMA1, JIMT1, CAL51, MCF7, HCC1806, MDAMB231, HS578T, HCC1395, HCC1937, HCC202), which suggested significant inhibition of survival in BC cells. Most CERES scores for other SLC39A genes in BC cell lines were > -1, which suggested that they were not a key genes for BC cell survival (Supplementary Table S1). To evaluate the association of SLC39A7 and BC cell growth. MCF7 and MDA-MB-468 cells were transfected with control siRNA or siR-SLC39A7. As shown in Figure 3B, the expression of SLC39A7 was significantly down-regulated with siR-SLC39A7 treatment in both MCF7 and MDA-MB-468 cells. CCK-8 assay was performed to detect the growth inhibition of siR-SLC39A7. As shown in Figure 3C, siR-SLC39A7 significantly decreased MCF7 and MDA-MB-468 cells growth over the time (*P*<0.001). To further certificate the results, the cell colony assay was performed. As shown in Figure 3D, siR-SLC39A7 significantly inhibited the number and size of cell colony compared with the control in MCF7 and MDA-MB-468 cells. These results suggested that SLC39A7 is a key gene for survival of BC cells.

### Up-regulated SLC39A7 is associated with intrinsic sub-types, TNM statues and recurrence of BC

**Figure 4:**
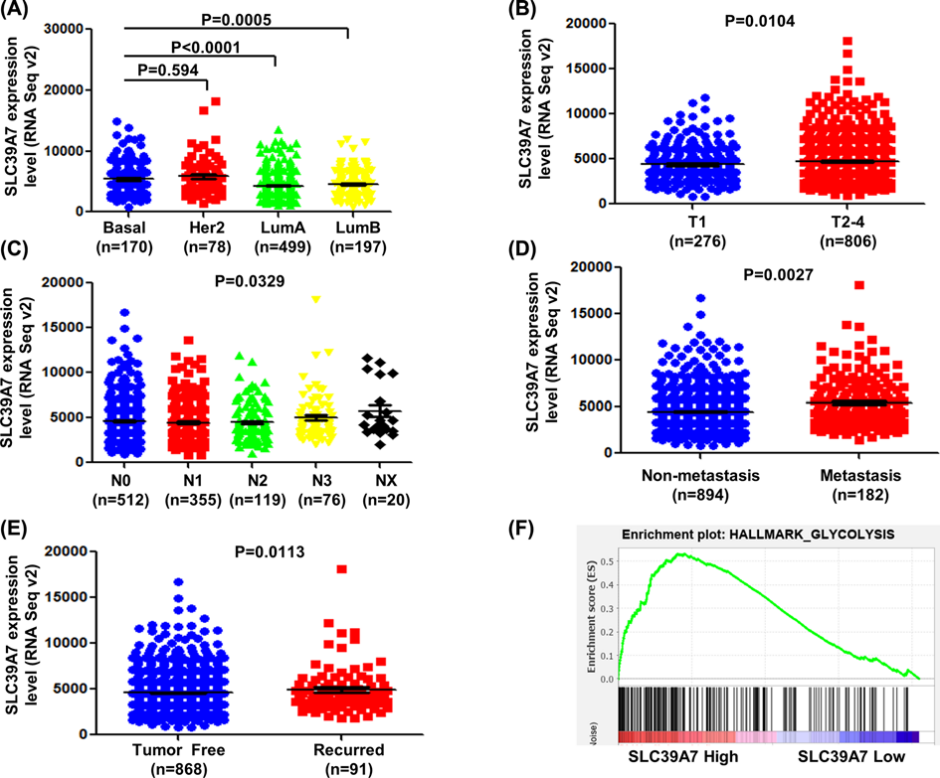
The association of SLC39A7 with advanced stage, metastasis and malignant degree of breast cancer was analyzed from TCGA_BRCA dataset (**A**) Correlation analysis was performed between expression level of SLC39A7 and intrinsic sub-types. (**B**) Comparison between different relative SLC39A7 mRNA levels in breast cancer patients with different tumor size. (**C**) Scatter plot of relative SLC39A7 mRNA levels in breast cancer patients with various metastatic lymphoma statues. (**D**) Comparison between SLC39A7 mRNA expression in breast cancer patients with metastasis or not. (**E**) Scatter plot of relative SLC39A7 mRNA level in breast cancer patients with and without recurrence was compared. (**F**) The gene signatures of glycolysis were highly enriched in patients with SLC39A7 high expression by GSEA of TCGA_BRCA dataset.

To investigate the role of SLC39A7 in BC progression, we analyzed the expression level of SLC39A7 in different intrinsic sub-types, TNM statues, and recurrence of patients with BC from TCGA. Totally, 1082 patients were included in the analysis. As shown in Figure 4A, the mRNA expression of SLC39A7 showed no significant difference between Basal group and HER2 group. In contrast, in LumA and LumB groups, the mRNA expression of SLC39A7 was significant lower than patients with Basal BC (*P*<0.05). T1-4 represents from primary tumor and closely related to tumor size and cell proliferation according to the criteria from the American Joint Committee on Cancer (AJCC). T1 means the tumor is small with minimally invasive within primary organ site. T2-4 means the tumor is larger than T1 with more seriously invasive within or beyond the primary organ site. SLC39A7 expression level was significantly increased in T2-4 stage compared with T1 stage BC tissues (Figure 4B, *P*=0.0104). “Metastasis” is defined as presence of distant locations or spread via blood way or lymphatics beyond. As shown in Figure 4C,D, SLC39A7 expression was significantly correlated with metastasis and spread through lymphatics pathway in patients with BC (*P*<0.05). “Disease free” is defined as disease free status since initial treatment. The expression of SLC39A7 was observed higher in recurrence group (Figure 4E, *P*<0.05) These results suggest that SLC39A7 was associated with BC progression and might have prognostic significance for patients with BC.

### The gene set enrichment analysis of SLC39A7 in BC

GSEA was performed to evaluate Hallmark effect gene sets. The patients with BC were divided into two groups according to the expression of SLC39A7. As shown in Table 5, all significant pathways of Hallmark effect gene sets were presented. The most valuable top 5 were glycolysis, DNA repair, unfolded protein response, reactive oxygen species pathway and MTORC1 pathway. The GSEA of glycolysis was shown in Figure 4F. These results suggested that SLC39A7 plays a vital role by regulating above classic cancer-related signaling pathways, which promotes cancer process.

**Table 5:** Gene sets enriched in the high SLC39A7 expression phenotype

Gene set name	NES	NOM *P*-value	FDR *q*-value
HALLMARK_GLYCOLYSIS	2.245	0	0.001
HALLMARK_DNA_REPAIR	1.967	0	0.026
HALLMARK_UNFOLDED_PROTEIN_RESPONSE	1.929	0	0.025
HALLMARK_REACTIVE_OXYGEN_SPECIES_PATHWAY	1.903	0	0.025
HALLMARK_MTORC1_SIGNALING	1.889	0.006	0.023
HALLMARK_OXIDATIVE_PHOSPHORYLATION	1.812	0.016	0.038
HALLMARK_MYC_TARGETS_V2	1.796	0.015	0.036
HALLMARK_MYC_TARGETS_V1	1.720	0.040	0.059
HALLMARK_PI3K_AKT_MTOR_SIGNALING	1.664	0.010	0.078
HALLMARK_CHOLESTEROL_HOMEOSTASIS	1.657	0.019	0.075
HALLMARK_UV_RESPONSE_UP	1.518	0.026	0.155
HALLMARK_ADIPOGENESIS	1.483	0.059	0.174
HALLMARK_PEROXISOME	1.428	0.046	0.215

Notes: Gene sets with NOM *P*-value < 0.05 and FDR *q*-value < 0.25 were considered as significantly enriched.

Abbreviations: FDR, false discovery rate; NES, normalized enrichment score; NOM, nominal.

### The protein expression of SLC39A7 in BC and prognosis analysis

**Figure 5:**
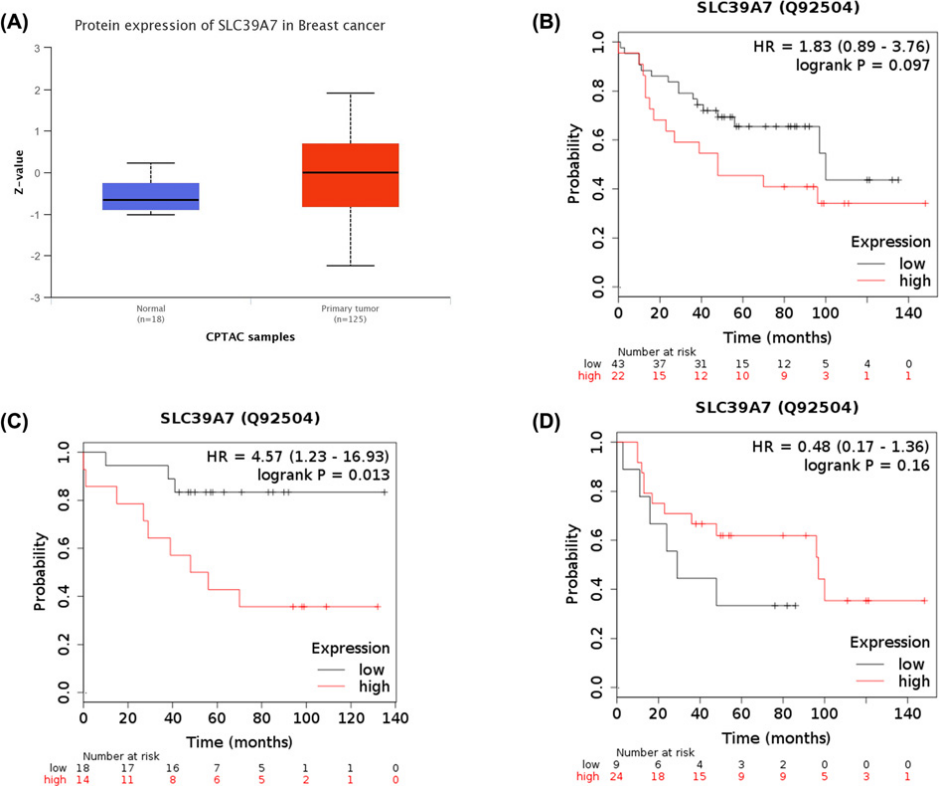
Protein expression and prognostic correlation of SLC39A7 in patients with BC (**A**) Protein levels of SLC39A7 in primary tumors and in corresponding normal tissues in patients with breast cancer by using Uaclan database. The correlation between SLC39A7 expression in protein level and overall survival of patients with breast cancer (**B**), ER-positive subtype (**C**), and ER-negative subtype (**D**) by using Kaplan–Meier plotter were shown.

The mRNA expression can’t perfectly represent the protein level. Hence, the protein expression of SLC39A7 in BC from CPTAC was analyzed by UALCAN database. As shown in Figure 5A, the protein level of SLC39A7 was significant higher in primary tumor tissue of BC than in normal tissues. Then, the OS was analyzed by using a Kaplan–Meier plotter. The SLC39A7 high expression was not associated with OS of patients with SLC39A7 low expression [HR = 1.83(0.89–3.76), *P*=0.097, Figure 5B]. Because SLC39A7 expression is associated with the OS of patients with LumA BC, the cases were divided into two groups according to the ER expression statues. High expression of SLC39A7 was significantly associated with worse OS in ER positive patients [HR = 4.57(1.23–16.93), *P*=0.013, Figure 5C], but no significant correlation was found between SLC39A7 expression and OS of patients with ER negative BC [HR = 0.48(0.17–1.36), *P*=0.16, Figure 5D]. These results were consistent with the above conclusion.

## Discussion

Intracellular and extracellular Zn levels and distributions were spatiotemporally regulated by the specific Zn transporters regulate in a tissue-specific manner [24]. Aberrant Zn transporters have been reported to be associated with specific diseases, including diabetes, Alzheimer’s disease, cancers, and other pathological processes [25–30]. Zn usually acts as a cancer suppressive agent, and SLC39 family 14 genes are highly expressed in various kinds of cancers. Low levels of Zn have been detected in serum and tumor tissues of patients with prostate cancer, and downregulated expression of SLC39A1, SLC39A2 and SLC39A3 has been demonstrated [31]. Zn levels in breast tissue and serum of patients with BC are higher and lower than which in healthy people, respectively [32]. Moreover, both clinical and basic studies have suggested the importance of the LIV-1 subfamily, such as SLC39A6, SLC39A7 and SLC39A10 in BC progression [33]. Hence, it’s necessary to explore the prognosis values of all SLC39A genes expression on patients with BC.

In the present study, we analyzed the mRNA expression profiles of all SLC39A genes in BC tissues and analyzed their correlations with overall survival outcomes in patients with BC by using available databases. Our results suggested that SLC39A1, SLC39A3, SLC39A4, SLC39A5, SLC39A6, SLC39A7, SLC39A9, SLC39A10, SLC39A11 and SLC39A13 were significantly higher in cancer tissue than which in normal tissue, whereas, SLC39A8 and SLC39A14 expressed higher in normal tissue. High expression of SLC39A2, SLC39A3, SLC39A4, SLC39A5, SLC39A7, SLC39A12 and SLC39A13 was significantly associated with worse OS in patients with BC. In contrast, high mRNA levels of SLC39A6 and SLC39A14 showed favorable OS. Through subgroup analysis, expression of all SLC39A members was correlated with prognoses of patients with specific BC. Moreover, we identified that SLC39A7 was a key gene in survival of BC, which is especially associated with LumA BC.

A line of evidences has demonstrated the importance of SLC39A members in the progression of BC [24]. Knockdown of SLC39A6 blocks intracellular homeostasis of Zn level, which leads to cell survival underlying a hypoxic environment [34]. Expression of SLC39A6 is reduced when promoting expression of epithelial–mesenchymal transition, which resulting in cell resistance to hypoxia [33,34]. Through these mechanisms, SLC39A6 high expression is associated with a longer relapse free survival period in patients with BC, which is consistent with our results. Interestingly, SLC39A10 was found higher expression in BC tissue, but not associated with the total OS of patients with BC. Previous studies have reported SLC39A10 mRNA is higher expressed in a metastatic than in a non-metastatic BC cell line. In addition, silencing SLC39A10 expression in the metastatic cell line inhibited both zinc intake and cell migration [35,36]. Our findings that high expression of SLC39A10 was associated with worse OS in patients with lymphoma-positive BC, but not correlated the OS in patients with lymphoma-negative BC suggested that SLC39A10 plays a vital role in metastatic BC. In addition, SLC39A10 high expression also associated the worse OS of patients in Basal, HER2 and combined endocrine therapy groups. High expression of SLC39A10 was associated with worse prognosis of gastric cancer and glioblastoma [37,38]. Studies have reported that SLC39A1 were expressed in prostate cancer and gastric cancer, which leaded zinc was depleted in adenocarcinomatous glands [39,40]. The association between SLC39A1 and BC is less reported previously. In the present study, SLC39A1 was highly expressed in BC samples, but not associated with the OS. Through subgroup analysis, we found that SLC39A1 high expression was linked with poorer OS in patients with LumB and HER2 BC. These results suggested that SLC39A1 might be tightly associated with HER2 expression of BC. In addition, high expression of SLC39A1 suggested a worse OS in poor differentiated BC patients, but a better OS in well differentiated type, which suggested a various molecular functions of which in BC cells. Similar results were observed in SLC39A9. SLC39A9 also portrayed as mAR that related to the extranuclear action of androgens in BC cells [41]. Chen et al. found that androgen and dihydrotestosterone (DHT) increased migration and invasion of nuclear androgen receptor (nAR)-negative bladder cancer cells via a newly identified membrane AR-SLC39A9-mediated intracellular signaling [42]. Hence, SLC39A9 may play a vital role in mAR-androgens signaling pathway and finally affect prognosis of patients with BC. In addition, SLC39A3, SLC39A4, SLC39A7 and SLC39A13 were also highly expressed in BC tissue and positively correlated with a worse OS in patients. SLC39A14-mediated zinc acculation in muscle progenitor cells represses the expression of class I myosin and myocyte enhancer factor 2C and prevents muscle-cell differentiation and induces myosin heavy chain loss, which leads metastatic cancer promoting cachexia [43]. In contrast, SLC39A14 was expressed lower in tumor sample, and higher expression of which indicated a favorable OS in patients with BC, which suggested different role of SLC39A14 in BC. All those members have a close correlation with some clinicopathological features. Even for SLC39A8, which had no differential expression, or not be significantly associated with the total OS of patients with BC, was positively associated with a better prognosis in LumB group.

By investigating the data from Project Achilles, we identified the SLC39A7 playing a vital role in cell survival of BC instead of other SLC39A members. Taylar et al. first reported the relationship between Zn and tamoxifen resistance therapeutic strategy and indicated that SLC39A7 is abundantly expressed in a BC cell line - tamoxifen-resistant MCF7 [44]. SLC39A7 is expressed in the endoplasmic reticulum (ER) membrane and activated with phosphorylation by casein kinase II (CK2). CK2 stimulates SLC39A7 activating which promotes Zn releasing from the ER into the cytoplasm and the released Zn inhibits phosphorylation of protein and increased the activity of tyrosine kinases, Akt survival and extracellular regulated kinase 1/2 growth signaling pathways, which promote cancer progression [45]. In the present study, SLC39A7 was highly expressed in BC tissues, and high expression of SLC39A7 suggested a worse prognosis of patients with BC. Through subgroup analysis, we found that highly expressed SLC39A7 was associated with OS of patients with LumA BC instead of other intrinsic sub-types, which suggested a tight link between SLC39A7 and ER. Furthermore, SLC39A7 was expressed lower in LumA and LumB intrinsic sub-types of BC than in Basal BC. The results on protein level also certificated these conclusions. Ectogenous Zn treatment could contribute to the activation of EGFR, Src and IGF-1R signaling molecules, which enhances growth and invasion of BC [46–48]. However, these activations could not be observed when down-regulating SLC39A7. In addition, knockdown of SLC39A7 inhibits migration of BC cells [44,49]. Our findings suggested that higher expressed SLC39A7 was associated with more serious tumor size and metastasis, which were consistent with the *in vitro* experiments [48]. In addition, we found that knockdown of SLC39A7 inhibited viability and colony of BC cells. Nimmanon et al. performed experiments and demonstrated that SLC39A7 -mediated zinc release from intracellular stores in controlling several pathways, including MAPK, mTOR and PI3K-AKT, involved in regulating cell survival and proliferation and often related to cancer process [50]. Through GSEA analysis, SLC39A7 was associated with several cancer-relative signaling pathways, which promotes cancer process. Through the above findings, SLC39A7 may be a potential therapeutic target for BC.

Two limitations were found in the present study. First, all expression and prognostic analysis were calculated by using the TCGA datasets. Lacking original clinical samples and data would limit the precise of last conclusion. Second, in Figure 5B, the result of SLC39A7 in protein level was consistent with the result of mRNA level. We speculated two major reasons may account for this point: (1) the size of samples in CPTAC was small, which lead the last result not accurate; (2) through a subgroup analysis according to the ER expression, we found that high expression of SLC39A7 was significantly associated with worse OS in ER positive patients, but no significant correlation was found between SLC39A7 expression and OS of patients with ER negative BC, which was consistent with the above results. It’s possible that large ER negative samples made the last results not significant.

In conclusion, our results suggested that SLC39A members were promising prognostic biomarkers of BC. The SLC39A7 plays a key role in growth and survival of BC cells. Importantly, our research on the findings of SLC39A members in BC is not insufficient, and suggests more basic and clinical studies focusing on their relationships.

## Supplementary Material

Supplementary Table S1

## Abbreviations

BC: breast cancer
CK2: casein kinase II
DHT: dihydrotestosterone
ER: endoplasmic reticulum
EUS-FNA: endoscopic ultrasound guided fine needle aspiration
HER2: human epidermal growth factor receptor 2
LumA: Luminal A
LumB: Luminal B
nAR: nuclear androgen receptor
OS: overall survival
PR: progesterone receptorprogesterone receptor
SLC: solute carrier
SLC39A: SLC family 39
ZIP: zinc importers
ZnT: zinc exporter
