# Supplementary material for: Analysis of the prognostic significance of solute carrier (SLC) family 39 genes in breast cancer
Source: Biosci Rep. 2020 Aug 13;40(8):BSR20200764. doi: 10.1042/BSR20200764 (PMC7426635; doi:10.1042/BSR20200764)
Supplement: Supplementary Table S1 [file BSR-2020-0764_supp.pdf]

Supplement Table 1. CERES dependence score of SLC family 39 genes in breast cancer cell lines.

Genes / Cell Lines	SLC39A1	SLC39A2	SLC39A3	SLC39A4	SLC39A5	SLC39A6	SLC39A7	SLC39A8	SLC39A9	SLC39A10	SLC39A11	SLC39A12	SLC39A13	SLC39A14
SKBR3	0.155938	0.065729	0.079799	-0.10851	-0.13942	0.003419	-1.45499	0.145226	-0.17535	-0.56442	-0.14143	0.055674	0.049709	-0.30232
MCF7	-0.03764	0.326776	0.00914	-0.12601	-0.16073	-0.07032	-1.25353	-0.26698	-0.12221	-0.23692	-0.1141	0.027601	-0.08407	-0.06941
KPL1	0.046469	0.239181	-0.1336	0.184978	-0.02311	-0.00279	-1.48884	-0.0141	-0.24661	-0.37874	-0.00418	0.148284	-0.44433	0.033252
ZR751	0.032958	0.078192	0.061482	0.220778	-0.09209	-0.59651	-0.90498	-0.13664	-0.03756	-1.0606	-0.05796	0.287633	-0.09737	-0.18056
HCC1187	-0.19853	0.219647	0.026553	0.01388	-0.43107	-0.22797	-0.55242	-0.10648	0.049091	-0.51303	0.619316	0.55828	0.024318	0.102018
HS578T	-0.0732	0.234696	0.145746	0.315179	-0.21617	0.028225	-1.16305	-0.03731	-0.05919	-0.30313	-0.18606	0.02932	-0.09983	-0.1735
HCC1937	0.003426	0.260761	0.16831	0.026271	-0.10929	-0.22731	-1.10719	-0.09986	-0.15492	-1.12189	-0.11978	0.248195	0.003964	-0.16474
AU565	0.162487	0.30641	0.274043	0.004593	-0.15841	-0.26334	-1.35005	0.15841	0.036211	-0.618	-0.15051	0.185193	0.078328	0.03901
DU4475	-0.05029	0.294494	0.304014	0.294861	0.072879	-0.21085	-0.98218	-0.24362	0.155466	-0.83459	0.125267	0.053511	-0.0357	0.229618
HCC38	0.082324	-0.04403	0.294727	0.148442	-0.17869	0.040245	-1.35411	-0.03866	-0.23884	-0.40621	0.166904	0.469244	-0.48421	-0.04316
HCC1419	0.022142	0.268064	0.276045	-0.04706	-0.02616	-0.49149	-1.64171	-0.21032	-0.08506	-0.81289	-0.04036	0.044677	-0.09202	-0.14975
BT549	-0.11692	0.173592	0.252768	0.209937	-0.17751	-0.13247	-1.41245	0.009561	0.016486	-0.32238	-0.12354	0.183765	-0.04801	0.09519
EFM19	0.066864	0.157786	0.236625	0.009393	-0.22644	-0.46898	-1.57816	-0.13769	-0.22337	-0.96338	-0.01794	0.33926	0.041746	-0.13339
HCC1428	0.22094	0.101523	0.22447	0.014989	-0.1112	-0.00933	-0.83781	-0.1946	0.010913	-0.30938	-0.09006	0.328222	-0.08355	-0.20641
HCC1143	-0.04626	0.346585	0.241744	0.201086	-0.0177	-0.20175	-1.44243	-0.13741	-0.14204	-0.70119	0.15516	0.242556	-0.28999	-0.15517
MDAMB436	-0.13105	0.23668	0.106023	0.175664	0.046836	-0.15832	-1.65513	-0.09327	-0.16993	-0.77941	0.061536	0.088986	-0.05188	-0.19204
MDAMB157	-0.00488	0.145984	0.17369	0.143505	-0.03414	-0.1635	-0.78699	-0.00237	-0.45708	-0.64561	-0.00403	0.153831	-0.08508	-0.07991
HCC1806	-0.01448	0.221106	0.011261	0.237084	-0.09676	-0.04791	-1.24296	-0.0022	0.019862	-0.63867	-0.03541	0.142488	-0.11615	-0.24188
HCC1395	0.051499	0.18205	0.147295	0.069926	0.063509	-0.07088	-1.12081	-0.027	-0.24023	-0.4796	-0.02208	0.145368	-0.08272	-0.11604
JIMT1	-0.07656	0.132622	0.120531	0.217382	-0.09106	-0.08976	-1.27045	-0.09287	-0.17517	-0.4364	-0.02202	0.166226	-0.23373	-0.16155
HMC18	-0.04571	-0.01892	0.11654	0.008856	-0.08468	-0.19928	-1.38176	-0.00831	-0.08263	-0.62228	0.045198	0.089758	0.002376	0.027938

HCC202	-0.15133	0.345004	0.155686	0.036054	0.090737	-0.82168	-1.01284	-0.23404	-0.0742	-1.23228	-0.17036	0.195494	-0.0555	-0.08365
MDAMB231	-0.39861	0.120791	0.138759	0.136288	-0.09128	-0.15327	-1.24216	-0.12514	0.035357	-0.85423	-0.00239	0.194545	-0.2632	0.026741
CAMA1	-0.00937	0.20661	-0.05791	-0.15777	-0.22995	0.01949	-1.31673	-0.12248	-0.10622	-0.48611	-0.04627	0.271118	-0.11172	-0.16001
MDAMB468	-0.00079	-0.00509	0.23944	0.074555	0.050767	-0.21967	-1.54929	-0.11453	-0.13132	-0.60718	-0.01289	0.009146	0.137481	-0.0969
CAL51	-0.08607	0.190979	0.04845	0.178742	0.0048	-0.18192	-1.27002	-0.06666	-0.08025	-0.351	0.007307	0.136524	-0.01004	-0.16427
HCC1954	0.132186	0.093088	0.04629	0.204573	0.033252	-0.07656	-1.47496	-0.12324	-0.05987	-0.43699	-0.01213	0.357336	-0.29608	0.030241
MDAMB415	-0.06462	0.273701	0.292406	0.206968	-0.08883	-0.09727	-0.59262	0.035298	-0.19042	-1.03034	0.001304	0.009749	0.056794	-0.17199
MDAMB453	-0.10472	0.276964	0.10404	-0.15832	-0.08773	-0.13563	-1.39533	-0.20475	0.133624	-0.59336	-0.03188	0.272197	-0.08218	-0.41491
SUM149PT	-0.10383	0.191956	-0.14456	-0.11708	0.196804	-0.23585	-1.48851	-0.11017	-0.08361	-1.16446	0.247549	-0.09926	0.019292	-0.35339
SUM159PT	-0.07966	0.215079	0.134659	0.046275	0.008684	-0.14745	-1.37168	0.092005	-0.15097	-0.63677	-0.13233	0.318632	-0.02749	-0.14717
SUM229PE	0.004314	0.267957	0.217084	-0.01704	-0.13957	-0.09666	-1.48738	-0.19204	0.269143	-0.24744	-0.09143	0.109729	0.052929	-0.16409
SUM52PE	0.435981	0.171199	0.158306	0.15737	0.166304	0.191308	-1.46112	-0.23862	-0.25344	-0.46008	0.0131	0.387684	0.020815	-0.30376
